# Acute Effects of Various Stretching Techniques on Range of Motion: A Systematic Review with Meta-Analysis

**DOI:** 10.1186/s40798-023-00652-x

**Published:** 2023-11-14

**Authors:** David George Behm, Shahab Alizadeh, Abdolhamid Daneshjoo, Saman Hadjizadeh Anvar, Andrew Graham, Ali Zahiri, Reza Goudini, Chris Edwards, Robyn Culleton, Carina Scharf, Andreas Konrad

**Affiliations:** 1 School of Human Kinetics and Recreation, Memorial University of Newfoundland, St. John’s, NL A1C 5S7 Canada; 2 Department of Sport Injuries and Corrective Exercises, Faculty of Sport Sciences, Shahid Bahonar University of Kerman, Kerman, 76169-13439 Iran; 3 Institute of Human Movement Science, Sport and Health, Graz University, Mozartgasse 14, 8010 Graz, Austria

**Keywords:** Flexibility, Stretch intensity, Stretch duration, Trained state, Sex, Age

## Abstract

**Background:**

Although stretching can acutely increase joint range of motion (ROM), there are a variety of factors which could influence the extent of stretch-induced flexibility such as participant characteristics, stretching intensities, durations, type (technique), and muscle or joint tested.

**Objective:**

The objective of this systematic review and meta-analysis was to investigate the acute effects of stretching on ROM including moderating variables such as muscles tested, stretch techniques, intensity, sex, and trained state.

**Methods:**

A random-effect meta-analysis was performed from 47 eligible studies (110 effect sizes). A mixed-effect meta-analysis subgroup analysis was also performed on the moderating variables. A meta-regression was also performed between age and stretch duration. GRADE analysis was used to assess the quality of evidence obtained from this meta-analysis.

**Results:**

The meta-analysis revealed a small ROM standard mean difference in favor of an acute bout of stretching compared to non-active control condition (ES = −0.555; Z = −8.939; CI (95%) −0.677 to −0.434; *p* < 0.001; I^2^ = 33.32). While there were ROM increases with sit and reach (*P*  = 0.038), hamstrings (*P* < 0.001), and triceps surae (*P*  = 0.002) tests, there was no change with the hip adductor test (*P* = 0.403). Further subgroup analyses revealed no significant difference in stretch intensity (*P*  = 0.76), trained state (*P*  = 0.99), stretching techniques (*P*  = 0.72), and sex (*P*  = 0.89). Finally, meta-regression showed no relationship between the ROM standard mean differences to age (R^2^ = −0.03; *P*  = 0.56) and stretch duration (*R*^2^ = 0.00; *P*  = 0.39), respectively. GRADE analysis indicated that we can be moderately confident in the effect estimates.

**Conclusion:**

A single bout of stretching can be considered effective for providing acute small magnitude ROM improvements for most ROM tests, which are not significantly affected by stretch intensity, participants’ trained state, stretching techniques, and sex.

**Supplementary Information:**

The online version contains supplementary material available at 10.1186/s40798-023-00652-x.

## Key Points

1. The meta-analysis on joint range of motion (ROM) increases revealed a small effect size in favor of an acute bout of stretching compared to the control condition.
2. Subgroup analysis revealed a significant increase in ROM with sit and reach, hamstrings, and triceps surae tests, but no improvement with the hip adductor tests. Whereas all moderating variables presented significant increases in ROM, further subgroup analyses revealed no significant difference in ROM gains with the stretch intensity, trained state of the participants, stretching techniques, and sex.
3. A meta-regression showed no relationship between the effect sizes to age and stretch duration, respectively.

**Supplementary Information:**

The online version contains supplementary material available at 10.1186/s40798-023-00652-x.

## Introduction

Recent commentaries [1, 2] and systematic reviews [3, 4] have revealed that other types of stretching and other activities such as resistance training and foam rolling may provide similar acute improvements in range of motion (ROM) as static stretching. Whereas other techniques such as foam rolling can acutely and chronically improve ROM [5–8], stretching within a pre-activity warm-up is still a predominant preparation activity [9–13]. The controversy regarding performance impairments associated with prolonged static stretching as a pre-event (warm-up) activity led to a paradigm shift toward dynamic over static stretching [9–13]. However, recent reviews [9, 12, 13] have highlighted the limitations of this body of research. They have suggested that since an acute increase in ROM may benefit some sports performance and contribute to a decreased incidence of musculotendinous injuries, especially with explosive and change of direction movements [14], appropriate durations (< 60-s per muscle group) [9–13] of static stretching would still be a beneficial component of a warm-up. But, are all forms of stretching within a single session an effective means of improving ROM acutely, which may contribute to positive influences on fitness, health, or preparation for training and competition?

Some of the stretching variables affecting acute changes in ROM or flexibility are the type of stretch technique, intensity, duration, as well as the sex and trained state of the individual [9, 15]. All the various types of stretching techniques such as static stretching (SS), dynamic (DS), ballistic stretching, proprioceptive neuromuscular facilitation (PNF), and others can increase ROM [9, 16]. While SS involves lengthening a muscle until either a stretch sensation or the point of discomfort is reached and then holding the muscle in a lengthened position for a prescribed period of time [10, 11, 17, 18], DS uses a controlled movement through the ROM of the active joint(s) [19]. Ballistic stretching differs from dynamic as it typically uses higher velocity movements with bouncing actions at the end of the ROM [20, 21]. There are two major forms of PNF stretching. The contract relax (CR) method involves a SS followed by an isometric contraction of the stretched muscle, with a subsequent stretch of the target muscle. The contract-relax-agonist contract method (CRAC) uses an additional contraction of the agonist muscle (i.e., opposing the muscle group being stretched) prior to an additional stretch of the target muscle [22, 23]. A number of studies suggest that PNF is more effective than SS or DS for improving ROM [24–26]. However, a recent meta-analysis reported greater ROMs achieved with SS over PNF training [27]. On the other hand, a number of studies report that a session of DS induced similar [28–30] or even greater [31, 32] ROM improvements than SS, while other articles show that an acute bout of SS is superior to DS [20, 33–36]. Thus, there is still no consistent clarity on whether there is a superior form of stretching to produce acute changes in ROM.

A number of acute stretching studies have shown that submaximal intensity stretches provide similar ROM benefits as near maximal point of discomfort stretches [37–41]. Apostolpoulos et al. [15] reviewed 79 articles mostly identified as low-quality studies with the objective to investigate the influence of stretch intensity on range of motion, delayed onset muscle soreness, and inflammation. With the lack of high-quality studies, the authors were unable to provide a definitive description regarding the impact of stretch intensity. Many of the stretching studies in their review did not describe the stretch intensity and those that did employ a wide variety of measures (e.g., point of discomfort, stretch to pain with the use of a therapist, maximum ROM with the use of a machine, therapist or a loaded stretch, maximum stretch with no pain). Cabido et al. [42] reported that the use of constant torque stretching (incremental increases in joint angle during the stretch) induced higher stretch intensities than constant angle stretches (maintaining the joint angle during the stretch). They found higher ROM and lower passive muscle stiffness with constant torque stretching (higher intensity) versus constant angle stretching. Fukaya et al. [43] also compared five studies that implemented constant torque or angle stretches and concurred that constant torque stretches produced a greater ROM [42, 44–47]. They also examined 12 other studies and found that higher stretch intensities provided greater ROM in six of those studies [48–53]. Furthermore, they reported that five higher stretch intensity studies resulted in greater decreases in passive muscle stiffness [48, 52, 54–56], while three other studies showed no significant passive stiffness differences between stretching intensities. Hence, the effects of higher stretch intensities during an acute bout of stretching have not been demonstrated to be consistently more effective than lower stretch intensities in these studies. It would be important to quantify whether painful or uncomfortable stretching intensities are necessary to obtain the greatest acute increases in joint ROM. This information needs to be updated to provide the most recent developments in the field.

Nearly, all stretching duration can ameliorate ROM [9]. The inclusion of control conditions in ROM studies is vitally important since just testing ROM (typical duration for a single test could be less than 5-s) will improve ROM [57]. Roberts and Wilson [58] reported that nine stretches of 5-s each provided similar increases in passive ROM as three stretches of 15-s; however, the 15-s stretches provided significantly greater active ROM than the 5-s stretches. On the other hand, a number of researchers have recommended SS for 30–60-s to optimally improve passive ROM. [59–61]. A meta-analysis by Thomas et al. [27] suggested a minimum SS duration of 5 min per week for each muscle group. Thus, there seems to be a relatively wide range of SS durations that can provide significant improvements in joint ROM.

Baseline measures of flexibility are commonly reported to be greater for women than men [62–68], which may be partially attributed to differences in muscle mass, joint geometry, and higher musculotendinous stiffness in men [9, 16, 69]. Hoge et al. [70] reported that following nine passive SS repetitions of 135-s each, ROM increased for the women but not for the men. Not all studies illustrate female flexibility superiority. Lopes-Minnaro et al. [71] reported similar male sit and reach flexibility as women; however, the women showed 8% greater pelvic flexion. While women tend to possess significantly greater intrinsic levels of flexibility, the relative effect of a single bout of SS may not be as disparate between sexes. Perhaps as men begin at a lower flexibility baseline, there is increased capacity for improvement. This relative effect needs further elucidation.

Therefore, the objective of this meta-analytical systematic review was to investigate the acute effects of stretching on ROM, with consideration of moderating variables such as stretching techniques, intensity, duration, as well as trained state or age of the participants, muscles tested, and sex.

## Methods

This review was conducted according to the 2020 PRISMA guidelines and the suggestions from Moher et al. [72] for systematic reviews with meta-analysis.

### Inclusion and Exclusion Criteria

In accord with PICOS (population, intervention, comparator, outcomes, study type) criteria, this review considered studies that investigated the intervention of an acute (single bout or session) effect of stretching on joint ROM (outcome) in healthy participants (population) compared to non-active control conditions (comparator). We included peer reviewed original studies published in English. The studies were included when they were either randomized controlled trials or controlled trials (type of studies). This implied that we excluded studies which were dealing with the training (chronic, long-term) effects of stretching, investigated any combined treatment (e.g., stretching combined with foam roller), or had another treatment as control condition (e.g., foam rolling). Moreover, we excluded review papers, case reports, special communications, letters to the editor, invited commentaries, conference papers, and theses.

### Search Strategy

An electronic literature search was performed in PubMed, Scopus, Web of Science, and SPORTDiscus. Papers were considered if they were published up to September 2022. Using AND and OR Boolean operators a systematic search was conducted using the following keywords: flexibility, “range of motion,” extensibility, and stretch*. In addition to the aforementioned keywords, the studies were filtered using the subsequent keywords to include controlled trials: “randomized controlled trial,” “controlled clinical trial,” randomized, placebo, randomly, and trial. Furthermore, to exclude animal studies ,we added a NOT operator with the following MeSH Term “exp animals/ not humans.” (Additional file 1). The systematic search was conducted by eight independent researchers (SA, AD, SH, AZ, RG, CE, CS, and AG). Initially, the articles were screened by their title and then abstract. If the content remained unclear, the full text was retrieved for further screening and identifying the relevant papers. Following this independent screening process, the researchers compared their findings. Disagreements were resolved by jointly reassessing the studies against the eligibility criteria.

### Extraction of the Data

From the included papers, the characteristics of the participants (i.e., age, trained state, sex), the sample size, the characteristics of the intervention (i.e., stretch per bout, stretch technique, stretch intensity, muscle stretched, muscle tested), and the results of the main variables (flexibility parameters) were extracted. For the flexibility parameters, pre- and post-intervention values plus standard deviations of the stretching and control groups were extracted. If some of the required data were missing in the included studies, the authors of the studies were contacted via email or similar channels (e.g., Research Gate). For studies with no available data, the corresponding authors were contacted. If no response was received from the corresponding authors, the studies were excluded.

### Risk of Bias Assessment

Egger’s regression intercept test and visual inspection of the funnel plot were applied to detect possible publication bias.

### Methodological Quality

The methodological quality of the included studies was assessed using the PEDro scale. In total, 11 methodological criteria were rated by eight independent researchers (SA, AD, SH, AZ, RG, CE, CS, and AG). A point was given if the study met the eligibility criteria and evidently a score of zero was assigned if the criteria was not satisfied. Hence, higher scores indicated better methodological quality of the study. In the case of conflict between the eight researchers, the methodological criteria were reassessed and discussed.

### Confidence in the Cumulative Evidence

Grading of Recommendations, Assessment, Development and Evaluations (GRADE) rating analysis was used to assess the quality of the outcomes by using the GRADEpro Guideline Development Tool software (gradepro.org). In general, GRADE has four levels of evidence quality: very low, low, moderate, and high. For GRADE analysis, six evaluation components were adopted (study design, risk of bias, inconsistency of results, indirectness, imprecision, and others [publication bias, large effect, plausible confounding, and dose response gradient]).

### Statistics and Data Synthesis

The meta-analysis was performed using Comprehensive Meta-Analysis software, according to the recommendations of Borenstein et al. [73]. By applying a random-effect meta-analysis, we assessed the effect size in terms of the standardized mean difference. If any study reported more than one effect size, the mean of all the outcomes (effect sizes) within one study was used for the analysis and was defined as combined (as suggested by Borenstein et al. [73]). Moreover, by applying a mixed-effect model, we performed subgroup analyses. Although there is no general rule of thumb [73], we only performed subgroup analyses when there were ≥ 3 studies included in the respective subgroups. Consequently, we performed subgroup analyses for the muscles tested (sit and reach vs. isolated hamstrings vs. triceps surae vs. hip adductors), intensity of stretch (i.e., high intensity vs. low intensity), trained state of the participants (active vs. sedentary), stretching techniques (static vs dynamic/ballistic vs. PNF), and sex (male vs female). To determine differences between the effect sizes of the subgroups, Q-statistics were applied [73]. Moreover, to assess possible relations in the moderating variables, we conducted a meta-regression (i.e., age of the participants, stretch duration) based on the recommendations of Borenstein et al. [73]. According to the recommendations of Hopkins et al. [74], the effects for a standardized mean difference of < 0.2, 0.2–0.6, 0.6–1.2, 1.2–2.0, 2.0–4.0, and > 4.0 were defined as trivial, small, moderate, large, very large, and extremely large, respectively. I^2^ statistics were calculated to assess the heterogeneity among the included studies, and thresholds of 25%, 50%, and 75% were defined as having a low, moderate, and high level of heterogeneity, respectively [75, 76]. An alpha level of 0.05 was defined for the statistical significance of all the tests. Data were presented in table and figure formats.

## Results

### Results of the Search

**Fig. 1:**
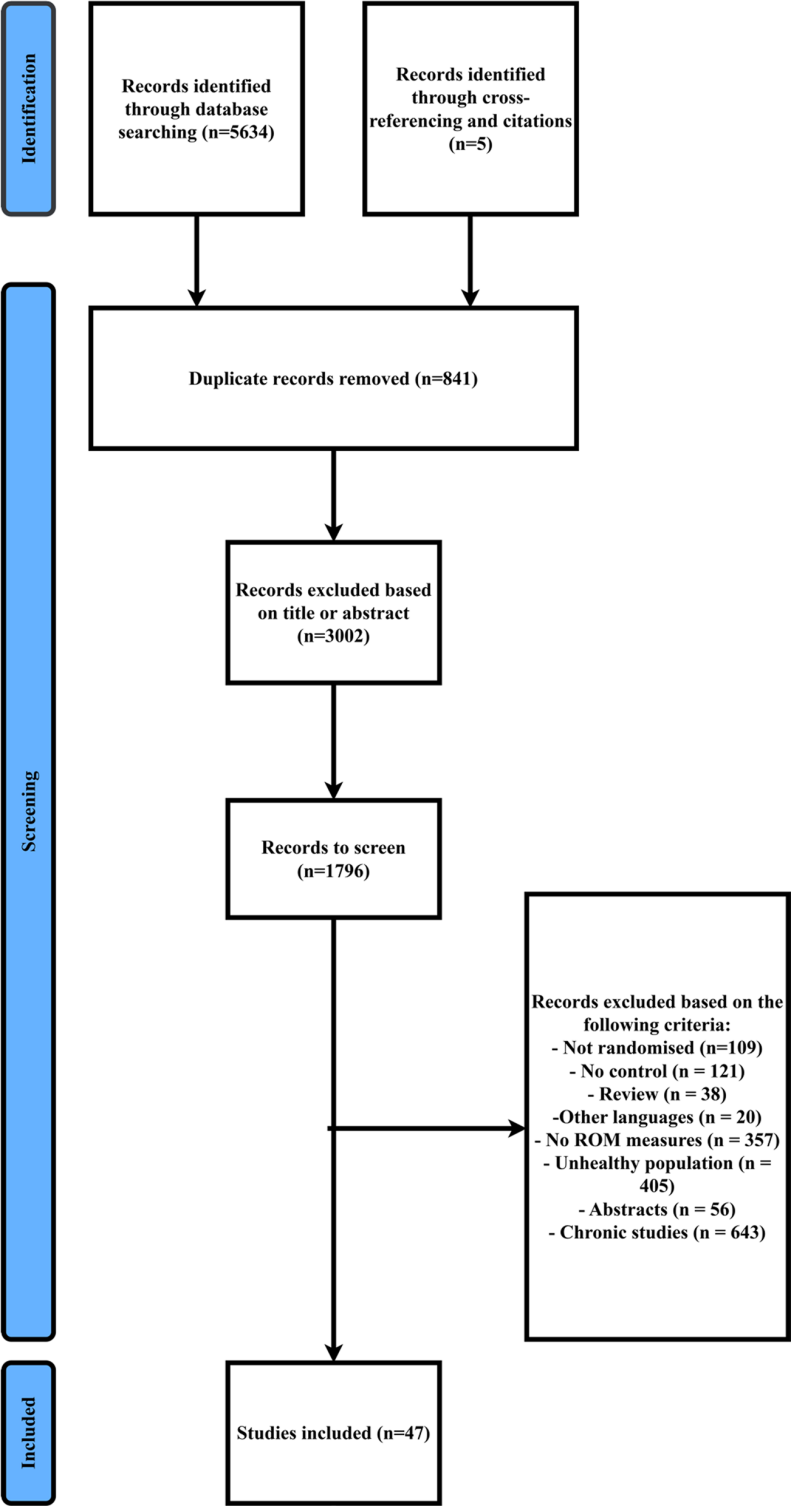
PRISMA flowchart

Overall, after removal of the duplicates, 4793 papers were screened, from which 42 papers were found to be eligible for this review (Table 1). After cross-referencing the included paper and their citation (via Google Scholar), of the 42 already included papers, five more papers were identified as relevant. Therefore, in total, 47 papers were included in this systematic review and meta-analysis. The search process is illustrated in the PRISMA flow diagram (Fig. 1). We have not cited the 4746 studies that were excluded as the reference list would be untenable.

**Table 1:** Study characteristics

Study	n	Age (years)	Sex	Trained State	Stretching Duration (s)	Stretched Muscle	Stretching Intensity	Outcome
Aguilar et al., 2012[93]	30	23	Mi	RA	600	Hamstrings	Below Maximum	Active and passive Hamstrings and hip flexors flexibility
Azevedo et al., 2011[94]	40	22.6	M	N/M	32	Hamstrings	Below Maximum	Hamstrings flexibility
Bacurau et al., 2009[20]	14	23.1	F	RA	540 & 1200	Quadriceps and Hamstrings	NR	Ballistic and static SR and hip flexors flexibility
Barbosa et al., 2018[95]	30	22.17	M	RA	180	Hamstrings	Below Maximum	Hams flexibility
César et al., 2016[96]	15	31.33	M	RA	90	Forearm Muscles	Maximum	Wrist hyperextension ROM
Chatzopoulos et al., 2019 [97]	24	15.08	F	RA	12 & 24 & 36	Hamstrings	NR	Active Hamstrings flexibility
Chen et al., 2013[98]	18	23.9	M	RA	210	Hamstrings	Below Maximum	Passive SLR
Lo et al., 2021[99]	20	20.7	Mi	E/P	150	Shoulder	Below Maximum	Shoulder internal rotation ROM
Coskunsu et al., 2021[100]	64	20.95	Mi	RA	120	Hamstrings	Maximum	Active SLR
Depino et al., 2000[101]	30	19.8	M	RA	120	Hamstrings	NR	Active Hamstrings flexibility
Espejo-Antunez et al., 2016[102]	42	21.5	Mi	RA	60	Hamstrings	Below Maximum	Passive Hamstrings flexibility
Hatano et al. 2022[91]	32	21.2	Mi	RA	300	Hamstrings	Below Maximum& Maximum	Passive Hamstrings flexibility
Hammer et al., 2017[103]	80	24.5	Mi	RA	120	Hip abductors	Below Maximum & Maximum	Hip abduction ROM
Hanney et al., 2017[104]	68	23.6	Mi	N/M	60	Trapezius	Maximum	Cervical ROM
Ikeda and Ryushi., 2019[105]	10	22	M	RA	900	Triceps surae	Below Maximum	Passive Dorsiflexion ROM
Kaneda et al., 2020[106]	17	23.2	M	S/U	120	Hamstrings	NR	Passive SLR ROM & Hamstrings flexibility
Konrad et al., 2017[45]	122	23.55	Mi	S/U	120	Triceps surae	Maximum	Dorsiflexion ROM
Konrad et al., 2019[107]	14	26.2	Mi	N/M	300	Triceps surae and Plantar Flexors	Maximum	Dorsiflexion ROM
Kuruma et al. 2013[108]	20	21	Mi	N/M	NR	Quadriceps	NR	Active and static Quadriceps flexibility
Lim et al., 2014[109]	32	22.5	M	S/U	28 & 30	Hamstrings	NR	Active Hamstrings flexibility
Maeda et al., 2016[110]	40	23.9	Mi	RA	120	Triceps surae	NR	Passive Dorsiflexion ROM
Maeda et al., 2017[111]	20	22.8	M	RA	120	Triceps surae	Maximum	Passive maximum DF ROM
Maeda et al., 2021[112]	30	23.5	M	N/M	300	Plantar Flexors	Maximum	Dorsiflexion ROM
Melo et al., 2021[113]	41	24.25	M	N/M	90	Hamstrings	Below Maximum & Maximum	Passive and active Hamstrings flexibility
Michaeli et al., 2017[114]	40	22.1	Mi	N/M	240	Hamstrings	NR	Static and dynamic SLR
Nishikawa et al., 2015[115]	108	20.3	Mi	RA	30	Hamstrings	Maximum	Active and passive Hamstrings flexibility
O'Hora et al., 2011[85]	30	25	Mi	N/M	30	Hamstrings	Maximum	Hams flexibility
Pepper et al., 2021[116]	20	25.6	Mi	S/U	66	Hip adductors	Maximum	Passive hip adduction ROM
Pollard and Ward 1997[117]	40	NR	NR	N/M	20	Hamstrings	NR	Passive SLR
Pratt & Bohannon, 2003[118]	24	24.7	Mi	N/M	180	Gastrocnemius	NR	Passive Dorsiflexion ROM
Rodrigues et al., 2017[119]	24	22.1	M	S/U	120	Hamstrings	NR	Passive Hamstrings flexibility
Rowlett et al., 2019[120]	20	26.1	Mi	N/M	180	Gastrocnemius and Soleus	NR	Passive Dorsiflexion ROM
Rubini et al., 2011[121]	30	28.5	F	RA	360	Hip adductors	NR	Hip adductors flexibility
Rubley et al., 2011[122]	33	20.6	Mi	RA	90	Hamstrings	NR	Sit and reach
Ryan et al., 2014[123]	52	22.2	M	RA	360	Lower Extremity	Maximum	Dynamic Sit and Reach
Schuback et al., 2004[124]	26	35.8	Mi	N/M	120	Hamstrings	Maximum	Passive hip flexion ROM
Silva et al., 2012[125]	20	24	M	S/U	120	Hamstrings	Maximum	Active hip flexion ROM
Smith et al., 2018[126]	58	22	Mi	RA	900	Lower Extremity	NR	Dynamic Sit and Reach
Spernoga et al., 2001[127]	30	18.8	M	RA	300	Hamstrings	Below Maximum	Active Hamstrings flexibility
Vernette-Santana et al., 2015[128]	34	23	NR	RA	96	Hamstrings	NR	Passive and active SLR
Viveiros et al., 2004[129]	10	25	Mi	S/U	10 & 30 & 60 & 120 & 180 & 360	Posterior Deltoid	Maximum	Shoulder extension ROM
de Weijer et al., 2003[130]	28	25.4	Mi	N/M	90	Hamstrings	Below Maximum	Active Hamstrings flexibility
Wiemann & Hahn, 1997[131]	57	27	M	S/U	45	Hamstrings	NR	Static and ballistic hip flexion ROM
Yildiz et al., 2020[132]	35	23.6	M	RA	200	Calf, Quadriceps, Hip adductors, Hamstrings, Hip Rotators	Maximum	Sit and reach
Young et al. 2006[40]	20	22.8	Mi	RA	60 & 120 & 240	Triceps surae	Below Maximum & Maximum	Static Dorsiflexion ROM
Zakas et al., 2003[133]	47	14.7	Mi	RA	300	Hip adductors, Hamstrings, Quadriceps, Gastrocnemius, Hip Flexors, Spinal Extensor	Maximum	Passive hip flexion and extension, knee flexion, dorsiflexion, and trunk flexion ROM
Zito et al., 1997[134]	19	25.4	Mi	N/M	30	Gastrocnemius	Maximum	DF ROM

DF: dorsiflexion, F: female, M: male, Mi: mixed group of males and females, NR: not reported, ROM: range of motion, SLR: straight leg raise, SR: sit and reach, RA: recreationally active, S/U: sedentary or untrained,

Overall, 110 effect sizes could be extracted from 47 eligible studies. In summary, 1658 participants with a mean age of 23.2 (± 3.4 years) participated in the included studies. Table 1 presents the characteristics and outcomes of the 47 studies.

### Risk of Bias Assessment and Methodological Quality

**Fig. 2:**
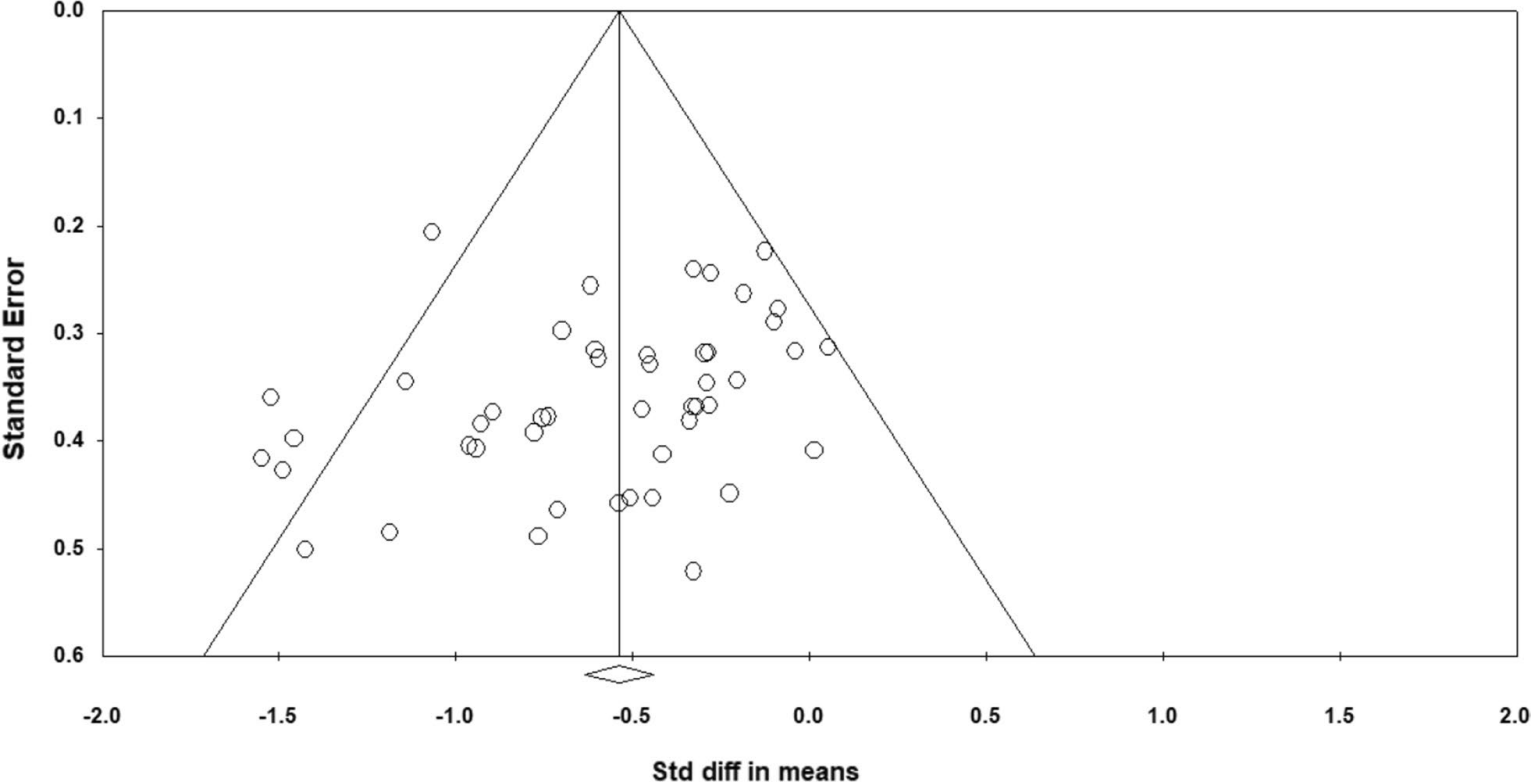
Funnel plot analysis. Std diff = Standard difference

Figure 2 shows the funnel plot, including all 47 studies in this meta-analysis. A visual inspection of the funnel plot and the Egger’s regression intercept test (intercept -1.597; P = 0.04) indicated reporting bias. The methodological quality, as assessed with the PEDro scale, revealed a range of scores between 5 and 9 points (out of 11) for all the included studies. The average PEDro score value was 7.1 (± 0.9) (median and mode values = 7), indicating a low risk of bias [77, 78] (Additional file 2). The assessors agreed with 100% out of the 517 criteria (47 studies × 11 scores). The mismatched outcomes were discussed, and the assessors agreed on the scores presented in Table 1.

### Confidence in Cumulative Evidence

For the study design, we have included randomized trials for the GRADE analysis. Risk of bias, indirectness, inconsistency, and imprecision showed no serious shortcomings. However, risk of bias assessment of the eligible studies showed publication bias as well as there was no large effect, no plausible confounding, and no dose response gradient. As a consequence, the analysis showed that we can be moderately confident in the effect estimates. This implies that the true effect is likely to be close to the estimate of the effect.

### Overall Effects

**Fig. 3:**
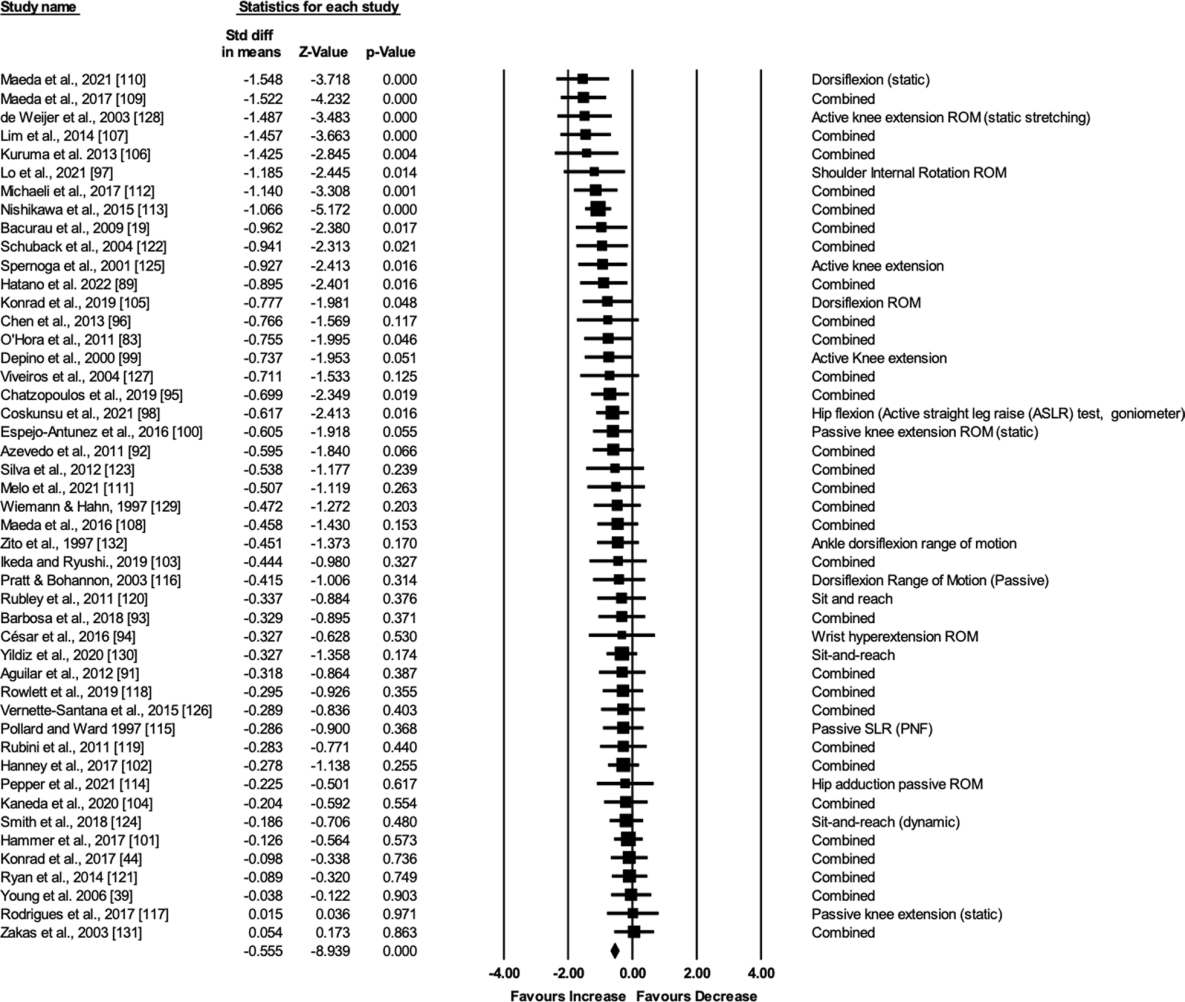
Forest plot presenting the 47 included studies investigating the acute effects of a single bout or session of stretching on range of motion (ROM). Std diff in means = standardized difference in means; CI = confidence interval; combined = mean of the selected outcomes of one study. ROM: range of motion, SLR: straight leg raise, PNF: proprioceptive neuromuscular facilitation

The meta-analysis on joint ROM revealed a small effect size in favor of stretching compared to the control condition (ES = −0.555; *Z* = −8.939; CI (95%) −0.677 to −0.434; *p* < 0.001; *I*^2^ = 33.32). Figure 3 presents the forest plot of the meta-analysis, sorted by the standard difference in means beginning with the lowest value (−1.548) up to the highest value (0.054).

### Moderating Variables

QA summary of all the subgroup analyses is provided in Table 2. The subgroups analyzed were the muscles tested (sit and reach (hamstrings and lower back) vs. isolated hamstrings vs. triceps surae vs. hip adductors), intensity of stretch (i.e., high intensity vs. low intensity), trained state of the participants (active vs. sedentary), stretching techniques (static vs dynamic/ballistic vs. PNF), and sex (male vs female).

**Table 2:** Statistics of the subgroup analysis. Negative values of Std diff (= standardized difference) in means indicate a favorable effect for stretching (and vice versa) on range of motion

Subgroup	Number of measures	Std diff in means (95% CI)		P Value	Q-statistics
*Muscles tested*					
Sit and Reach	5	−0.272	(−0.529 to −0.016)	0.038^a^	
Hamstrings	25	−0.674	(−0.831 to −0.518)	< 0.001^a^	
Triceps surae	11	−0.504	(−0.821 to −0.187)	0.002^a^	
Hip Adductors	4	−0.128	(−0.429 to 0.172)	0.403	
*Overall*	*45*	*−0.495*	*(−0.609 to −0.381)*	< *0.001*	(Q = 13.67; df (Q) = 3; P = 0.003)^b^
*Intensity of stretch*					
High intensity	20	−0.570	(−0.782 to −0.358)	< 0.001^a^	
Low intensity	13	−0.524	(−0.735 to −0.312)	< 0.001^a^	
*Overall*	*33*	*−0.547*	*(−0.697 to −0.397)*	< 0.001	(Q = 0.092; df (Q) = 1; P = 0.76)
*Trained state*					
Active	24	−0.496	(−0.655 to −0.337)	< 0.001^a^	
Sedentary	9	−0.499	(−0.825 to −0.173)	0.003^a^	
*Overall*	*33*	*−0.496*	*(−0.639 to −0.353)*	< *0.001*	(Q = 0.000; df (Q) = 1; P = 0.98)
*Stretching techniques*					
Static	36	−0.570	(−0.724 to −0.416)	< 0.001^a^	
Ballistic/Dynamic	10	−0.447	(−0.718 to −0.177)	0.001^a^	
PNF	*9*	−0.581	(−0.843 to −0.318)	< 0.001^a^	
*Overall*	*55*	*−0.548*	*(−0.667 to −0.429)*	< *0.001*	(Q = 0.667; df (Q) = 2; P = 0.72)

Sex					
Male	16	−0.607	(−0.853 to −0.361)	< 0.001^a^	
Female	3	−0.639	(−1.033 to −0.246)	0.001^a^	
*Overall*	*19*	*−0.616*	*(−0.825 to −0.408)*	< *0.001*	(Q = 0.019; df (Q) = 1; P = 0.89)

^a^ = significant difference within a group^b^ = significant difference between groups

Q-statistics of the subgroup analysis revealed a significant difference for the muscles tested (*P*  = 0.003). While there was an increase in ROM with the sit and reach test, hamstrings test, and triceps surae tests, no such change was seen in the hip adductor tests. Further subgroup analyses revealed no significant difference in the Q-statistics for the stretch intensity (*P*  = 0.76), trained state of the participants (*P* = 0.99), stretching techniques (*P*  = 0.72), and sex (*P*  = 0.89). Furthermore, meta-regression showed no relationship between the effect sizes to age (*R*^2^ = −0.03; *P* = 0.56) and stretch duration (R^2^ = 0.00; *P*  = 0.39), respectively.

## Discussion

The major finding of this meta-analysis was a small magnitude effect size ROM increase with acute stretching compared to control conditions. GRADE analysis showed that we can be moderately confident in the effect estimates. The stretching-induced acute small magnitude increase in ROM is in accord with prior reviews that have reported that all four forms of stretching (SS, DS, ballistic, and PNF) can increase joint ROM [10, 22, 79]. Behm et al. [12] reported an overall 8.04% (Cohen’s d = 0.55) ROM increase from 27 SS studies, whereas Radford et al. [80] in their review of five studies concluded that plantar flexor muscle stretching induced small but significant increases in ankle dorsiflexion. Underlying acute stretching mechanisms have been attributed to an increased stretch (pain) tolerance [41, 112], decreased muscle stiffness [45, 49, 51, 109] thixotropic effects (decreased tissue viscoelasticity) [42], muscle spindle dysfacilitation (primarily with prolonged SS), pre-synaptic inhibition (as evidenced by reduced Hoffman reflexes) [81], and fascicle rotation [9, 10, 82].

### Stretching Technique

The present review did not find any ROM differences based on the stretching technique. There are diverse reports indicating greater [83–85] or similar ROM increases with PNF vs. SS [86, 87] as well as similar [28, 29] or greater [31, 32] increases in flexibility with DS vs. SS. These findings contrast with other studies reporting that DS was not as effective for increasing ROM as SS [20, 33–36]. When examining 55 effect sizes (SS: 36, ballistic/DS: 10, PNF: 9 studies) with disparate stretch intensities, durations and other prescription components, the main message from this review is that all forms of stretching are similarly effective in promoting acute increases in joint ROM within a general population.

### Stretch Duration

Whereas the included studies with these 55 effect sizes used a wide variety of stretch durations, there was no significant difference in ROM gains based on stretch duration. ROM can be augmented with stretch durations as short as 5-s [57]. Nine stretches of 5-s induced similar increases in passive ROM as three stretches of 15-s; however, the longer duration stretches provided significantly higher active ROM than the shorter duration stretches [58]. Johnson et al. [88] did not find any knee extension ROM differences whether participants trained with nine repetitions of 10-s or three repetitions of 30-s. A systematic review of four studies [58, 59, 89, 90] by DeCoster et al. [79] indicated that while a stretching bout of 30-s might be most effective, durations greater than 30-s do not provide an additional ROM advantage. Studies employing increased stretch repetitions with shorter durations provided similar ROM improvements [58, 90]. A number of studies have recommended 30–60-s of SS to optimally improve ROM. [59–61]. Thus, while there is a spectrum of stretch durations that can significantly increase joint ROM, the present analysis indicates that there is no single duration that provides a significant ROM gain advantage.

### Stretch Intensity

Similarly, using a high or low stretch intensity did not significantly modulate ROM gains. One of the difficulties in analyzing stretch intensity is the lack of consistency in the description of intensity (e.g., point of discomfort, stretch to pain, maximum ROM with the use of a machine to maintain constant torque or constant angle, maximum stretch with no pain). While Apostolpoulos et al. [15] reviewed 79 articles of mostly low-quality studies, they were not able to definitively judge the impact of stretch intensity on joint ROM. A number of acute stretching studies have shown that submaximal intensity stretches provide similar ROM benefits as near maximal point of discomfort stretches [37–41]. Two reviews [42, 43] reported that constant torque stretching (higher intensity) induced greater ROM and lower passive muscle stiffness than constant angle (lower intensity) stretching. In the Fukaya et al. [43] review, only six of 12 other studies reported greater ROM with higher stretch intensity, and only five of eight higher stretch intensity studies reported greater decreases in passive muscle stiffness. Hatano et al. [91] reported a positive correlation between stretching intensity and the degree of change in ROM and muscle stiffness. Hence, while there is some evidence illustrating greater effectiveness for improving ROM with higher intensity stretching, the results of the present review reflect the overall variability in the literature. While many coaches in sports necessitating extreme ROM like gymnastics, wrestling, and figure skating anecdotally are proponents of higher stretch intensities to attain these high ROMs, the present review of the population in general did not reveal a positive association. Furthermore, one must be cautious as high stretching intensity may exacerbate inflammation in chronic clinical conditions while improving the ROM of soft and connective tissue in therapeutic and athletic populations [15].

### Muscles Tested

An acute bout of stretching will increase ROM in most tests (i.e., sit and reach (hamstrings and lower back), isolated hamstrings, and triceps surae ROM tests) with the exception of hip adductor [114, 119] and abductor [101, 131] ROM tests. Changes in hip adduction and abduction may be more limited by the skeletal configuration of the acetabulum inhibiting ROM increases to a greater degree than other joint movements with greater excursions. Furthermore, to limit motion and prevent dislocations, the thickness and volume of connective tissue is more extensive at the hip to maintain joint integrity during weight bearing movements, as compared to other joints such as the shoulder, which has greater range of motion and is not often not weight bearing. In addition, the hip adductor and abductor muscles commonly do not experience as expansive a ROM with activity as the hip flexors or extensors (e.g., with sprinting, jumping, bounding), and thus, the hip adductors and abductors might be less sensitive to increases in ROM. Finally, when considering the subgroup analysis of the respective muscles, it has to be noted that only two effect sizes each for the hip adductors and abductors were included, and hence, caution has to be taken not to overemphasize the results found.

### Participant Characteristics

The trained state, age, or sex of the participant did not present significant differences in ROM gains. Similarly, a recent meta-analysis comparing the effects of stretching and foam rolling on ROM reported no significant differences between participants’ age groups, activity levels, tested muscle by the ROM test (hamstrings, quadriceps, triceps surae, deltoid), stretch or foam rolling duration, sex, stretching technique (SS, DS), and the study design (parallel design, crossover) [6]. Furthermore, another meta-analysis examining crossover and non-local effects on ROM from unilateral, acute, passive, static stretching showed moderate magnitude increases in non-local (non-stretched) joint ROM in healthy young adults with no significant differences between trained state, stretching intensity, and sex [76]. Although stretching duration did not demonstrate significant differences in this Behm et al. meta-analysis [76], more than 240-s of stretching exhibited large magnitude increases in non-local ROM compared to only moderate magnitude improvements with lower (< 240- and < 120-s) stretching durations.

An initial thought might contend that the lower baseline levels of flexibility with untrained individuals would give them a greater training capacity for ROM improvements. However, when considering the capacity for extreme improvements in ROM seen with certain athletes (e.g., figure skaters, gymnasts, divers, contortionists), the extent of change is capacious. Thus, even with higher baseline flexibility, trained individuals still have extensive potential for increased ROM that would not differentiate them from untrained individuals with a single (acute) session of stretching [92].

Although older individuals tend to exhibit more restricted ROM [25, 84], relative increases in ROM with stretch training have been reported to be similar to younger adults [84], and they demonstrate greater degrees of flexibility than untrained older adults [84]. Moreover, women tend to have greater joint ROM than men [62–67] due to differences in muscle mass, joint geometry, and the degree of collagen in the musculotendinous unit [16]. Hence, the present results suggest that even when the baseline flexibility is more limited in untrained young or older adults or males compared to females, the potential for acute ROM increases is not hindered by age, sex, or trained state.

### Limitations

Limitations in the research included that not all muscle groups are equally represented in the literature, and further research should expand the scope of muscles tested as for example the limited research on hip adductors. Almost every study described in this review recruited young adults, and only two studies focused solely on females; hence a wider spectrum of participants needs to be investigated.

## Conclusions

This systematic review and meta-analysis demonstrated a small magnitude increase in ROM with stretching compared to the control condition. Acute increases in ROM occurred with all muscles tested (sit and reach, hamstrings, and triceps surae tests), but no improvement with the hip adductor tests, which might be attributed to more natural anatomical and functionally restricted movement patterns. There was also no significant difference in ROM gains with the stretch intensity, duration, trained state of the participants, stretching techniques, age, or sex suggesting relative acute increases in ROM possess a broad capacity for acute improvement. Consequently, it can be suggested that all types of stretching can be implemented acutely for diverse populations (i.e., male and female, trained and untrained individuals) with similar results expected.

### Supplementary Information

**Additional file 1.** Search Strategy.

**Additional file 2.** Table S1: PEDro scales.

## Data Availability

All data will be made available on request to the corresponding author.

## Abbreviations

CRAC: Contract-relax-agonist contract
DS: Dynamic stretching
ES: Effect size
ROM: Range of motion
PNF: Proprioceptive neuromuscular facilitation
SS: Static stretching
