# Supplementary material for: Acute Effects of Various Stretching Techniques on Range of Motion: A Systematic Review with Meta-Analysis
Source: Sports Med Open. 2023 Nov 14;9:107. doi: 10.1186/s40798-023-00652-x (PMC10645614; doi:10.1186/s40798-023-00652-x)
Supplement: Supplementary file 2 — Additional file 2. Table S1: PEDro scales. [file 40798_2023_652_MOESM2_ESM.docx]

Table 1: PEDro scales

| **Study** | **1** | **2** | **3** | **4** | **5** | **6** | **7** | **8** | **9** | **10** | **11** | **Total** |
| --- | --- | --- | --- | --- | --- | --- | --- | --- | --- | --- | --- | --- |
| Aguilar et al., 2012 [91] | Y | Y | N | Y | N | N | N | Y | Y | Y | Y | 7 |
| Azevedo et al., 2011 [92] | Y | Y | Y | N | N | N | Y | Y | Y | Y | Y | 8 |
| Bacurau et al., 2009 [19] | N | Y | N | Y | N | N | N | Y | Y | Y | Y | 6 |
| Barbosa et al., 2018 [93] | Y | Y | N | Y | Y | N | N | N | Y | Y | Y | 7 |
| César et al., 2016 [94] | Y | Y | N | Y | N | N | N | Y | Y | Y | Y | 7 |
| Chatzopoulos et al., 2019 [95 | Y | Y | N | Y | N | N | Y | Y | Y | Y | Y | 8 |
| Chen et al., 2013 [96] | Y | Y | N | Y | N | N | N | Y | Y | Y | Y | 7 |
| Coskunsu et al., 2021 [98] | Y | Y | N | Y | N | Y | Y | Y | Y | Y | Y | 9 |
| Depino et al., 2000 [99] | Y | Y | N | Y | N | N | N | Y | Y | Y | Y | 7 |
| Espejo-Antunez et al., 2016 [100] | Y | Y | N | Y | N | N | N | Y | Y | Y | Y | 7 |
| Hatano et al. 2022 [89] | Y | Y | N | Y | N | N | N | Y | Y | Y | Y | 7 |
| Hammer et al., 2017 [101] | Y | Y | N | Y | N | N | N | Y | Y | Y | Y | 7 |
| Hanney et al., 2017 [102] | Y | Y | Y | Y | N | N | Y | Y | Y | Y | Y | 9 |
| Ikeda and Ryushi 2019 [103] | N | Y | N | Y | N | N | N | Y | Y | Y | Y | 6 |
| Kaneda et al., 2020 [104] | Y | N | N | Y | N | N | N | Y | Y | Y | Y | 6 |
| Konrad et al. 2017 [44] | N | Y | N | Y | N | N | N | Y | Y | Y | Y | 6 |
| Konrad et al., 2019 [105] | Y | N | N | Y | N | N | N | Y | Y | Y | Y | 6 |
| Kuruma et al. 2013 [106] | Y | Y | N | Y | N | N | N | Y | Y | Y | Y | 7 |
| Lim et al., 2014 [107] | Y | Y | N | Y | N | N | Y | Y | Y | Y | Y | 7 |
| Lo et al., 2021 [97] | Y | Y | N | Y | N | N | N | Y | Y | Y | Y | 7 |
| Maeda et al., 2017 [108] | Y | Y | N | Y | N | N | N | Y | Y | Y | Y | 7 |
| Maeda et al., 2021 [109] | Y | Y | N | Y | N | N | N | Y | Y | Y | Y | 7 |
| Melo et al., 2021 [111] | Y | Y | Y | Y | N | N | Y | Y | Y | Y | Y | 8 |
| Michaeli et al., 2017 [112] | Y | Y | Y | Y | N | N | Y | Y | Y | Y | Y | 9 |
| Nishikawa et al., 2015 [113] | Y | Y | N | Y | N | N | N | Y | Y | Y | Y | 7 |
| O'Hora et al., 2011 [83] | Y | Y | N | Y | N | N | N | Y | Y | Y | Y | 7 |
| Pepper et al., 2021 [114] | Y | Y | Y | Y | N | N | Y | Y | Y | Y | Y | 9 |
| Pollard and Ward 1997 [115] | Y | Y | N | Y | N | N | Y | Y | Y | Y | Y | 8 |
| Pratt & Bohannon, 2003 [116] | Y | Y | N | Y | N | N | N | Y | Y | Y | Y | 7 |
| Rodrigues et al., 2017 [117] | N | N | N | Y | N | N | N | Y | Y | Y | Y | 5 |
| Rowlett et al., 2019 [118] | Y | Y | N | Y | N | Y | Y | Y | Y | Y | Y | 9 |
| Rubini et al., 2011 [119] | Y | Y | N | Y | N | N | N | Y | Y | Y | Y | 7 |
| Rubley et al., 2011 [120] | Y | Y | N | Y | N | N | N | Y | Y | Y | Y | 7 |
| Ryan et al., 2014 [121] | N | Y | N | Y | N | N | N | Y | Y | Y | Y | 6 |
| Schuback et al., 2004 [122] | Y | Y | Y | Y | N | N | N | Y | Y | Y | Y | 8 |
| Silva et al., 2012 [123] | Y | Y | N | Y | N | N | N | Y | Y | Y | Y | 7 |
| Smith et al., 2018 [124] | Y | Y | N | Y | N | N | N | Y | Y | Y | Y | 7 |
| Spernoga et al., 2001 [125] | Y | Y | N | Y | N | N | N | Y | Y | Y | Y | 7 |
| Vernette-Santana et al., 2015 [126] | N | N | N | Y | N | N | N | Y | Y | Y | Y | 5 |
| Viveiros et al., 2004 [127] | Y | Y | N | Y | N | N | N | Y | Y | Y | Y | 7 |
| de Weijer et al., 2003 [128] | Y | Y | N | Y | N | N | Y | Y | Y | Y | Y | 8 |
| Wiemann & Hahn, 1997 [129] | Y | Y | N | Y | N | N | N | Y | Y | Y | Y | 7 |
| Yildiz et al., 2020 [130] | Y | Y | N | Y | N | N | N | Y | Y | Y | Y | 7 |
| Young et al. 2006 [39] | Y | Y | N | Y | N | N | N | Y | Y | Y | Y | 7 |
| Zakas et al., 2003 [131] | Y | N | N | Y | N | N | N | Y | Y | Y | Y | 6 |
| Zito et al., 1997 [132] | Y | Y | N | Y | N | N | Y | Y | Y | Y | Y | 7 |
| Mean |  |  |  |  |  |  |  |  |  |  |  | 7.1 |
| Median |  |  |  |  |  |  |  |  |  |  |  | 7 |
| Mode |  |  |  |  |  |  |  |  |  |  |  | 7 |

PEDro scale criteria. 1. Eligibility criteria were specifed. 2. Subjects were randomly allocated to groups (in a crossover study, subjects were randomly allocated an order in which treatments were received). 3. Allocation was concealed. 4. The groups were similar at baseline regarding the most important prognostic indicators. 5. There was blinding of all subjects. 6. There was blinding of all therapists/researchers who administered the therapy/protocol. 7. There was blinding of all assessors who measured at least one key outcome. 8. Measures of at least one key outcome were obtained from more than 85% of the subjects initially allocated to groups. 9. All subjects for whom outcome measures were available received the treatment or control condition as allocated or, where this was not the case, data for at least one key outcome were analysed by “intention to treat”. 10. The results of between-group statistical comparisons were reported for at least one key outcome. 11. The study provided both point measures and measures of variability for at least one key outcome Y yes, N no
